# The effectiveness of interventions in supporting self-management of informal caregivers of people with dementia; a systematic meta review

**DOI:** 10.1186/s12877-015-0145-6

**Published:** 2015-11-11

**Authors:** Judith G. Huis in het Veld, Renate Verkaik, Patriek Mistiaen, Berno van Meijel, Anneke L. Francke

**Affiliations:** Netherlands Institute for Health Services Research (NIVEL), Utrecht, The Netherlands; Department of Public and Occupational Health, EMGO Institute for Health and Care Research, VU University Medical Center, Amsterdam, The Netherlands; Department of Psychiatry, EMGO Institute for Health and Care Research, VU University Medical Center, Amsterdam, The Netherlands; Inholland University of Applied Sciences, Amsterdam, The Netherlands; Parnassia Psychiatric Institute, The Hague, The Netherlands

**Keywords:** Dementia, Self-management support, Informal caregivers, Systematic review

## Abstract

**Background:**

Informal caregivers of people with dementia are challenged in managing the consequences of dementia in daily life. The objective of this meta-review was to synthesize evidence from previous systematic reviews about professional self-management support interventions for this group.

**Methods:**

In March 2014, searches were conducted in PubMed, CINAHL, Cochrane Library, Embase and PsycINFO. The PRISMA Statement was followed. Interventions were grouped using Martin’s targets of self-management, covering 5 targets: *relationship with family, maintaining an active lifestyle, psychological wellbeing, techniques to cope with memory changes* and *information about dementia*. Using an evidence synthesis, the outcomes from the included interventions were synthesized and conclusions were drawn about the level of evidence for the effectiveness of interventions within each target.

**Results:**

Ten high-quality systematic reviews were selected. Evidence exists for the effectiveness of professional self-management support interventions targeting *psychological wellbeing* on stress and social outcomes of informal caregivers. In addition, evidence exists for the effectiveness of interventions targeting *information* on ability/knowledge. Limited evidence was found for the effectiveness of interventions targeting *techniques to cope with memory change* on coping skills and mood, and for interventions targeting *information* on the outcomes sense of competence and decision-making confidence of informal caregivers.

**Conclusions:**

Scientific evidence exists for the effectiveness of a number of professional self-management support interventions targeting *psychological wellbeing* and *information*. Health care professionals could take account of the fact that psycho-education was integrated in most of the self-management support interventions that were found to be effective in this meta-review. Furthermore, longer and more intensive interventions were associated with greater effects.

**Electronic supplementary material:**

The online version of this article (doi:10.1186/s12877-015-0145-6) contains supplementary material, which is available to authorized users.

## Background

Nowadays, self-management and self-management support are becoming more and more important. Besides the fact that health policies encourage people to self-manage for as long as possible [1], most people also prefer to keep control over their own life and health care. A commonly used definition of self-management in this context is “the individual’s ability to manage the symptoms, treatment, physical and psychosocial consequences and lifestyle changes inherent in living with a chronic condition” [2]. Self-management is not only a task for patients but also for informal caregivers. In people with dementia, self-management increasingly becomes the responsibility of the informal caregivers as the disease progresses. However, self-management often makes great demands on informal caregivers. Besides managing problems in the person with dementia, they also have to manage their own problems, such as concerns about the future and the daily burden of caregiving. This can have negative consequences for the psychological wellbeing of the informal caregiver [3] and may have an impact on the relationship with the person with dementia [4].

Managing well with the problems and consequences of dementia is challenging for informal caregivers, and professional support may be needed. Nurses, psychologists or other professionals can act as partners with the informal caregivers, by supporting them in their decisions and actions to manage the disease and its consequences in daily life [5]. What type of support or intervention should be provided by professionals to informal caregivers depends on how the informal caregivers are managing or where they feel the need for support. A logical way to distinguish different types of self-management support interventions is to categorize them according to the main target of the intervention. Martin, et al. [6] distinguish five self-management targets for persons with dementia: *1) relationship with family, 2) maintaining an active lifestyle, 3) psychological wellbeing, 4) techniques to cope with memory changes, and 5) information* about dementia. Since self-management by informal caregivers focuses first and foremost on the patient, the patient targets are also applicable when categorizing self-management support interventions aimed at informal caregivers.

In recent decades, many interventions have been developed to provide self-management support to informal caregivers of persons with dementia. Most of the time however, these interventions were labeled not as such as the concept of self-management has emerged relatively recently. Self-management support interventions were labeled for example as ‘psychosocial interventions’, ‘support interventions’ or ‘case management interventions’. Related to these wide variety of labels used for these interventions, until now there has been no insight into the level of evidence for the effectiveness of different types of self-management support interventions for informal caregivers of persons with dementia. Nevertheless, there were already a lot of relevant review papers. We therefore conducted a systematic meta-review, making use of the self-management support targets defined by Martin, et al. [6]. Additionally, we aim to identify participant and intervention characteristics that are related to positive outcomes of self-management interventions.

- The primary question of this systematic meta-review is:
  What scientific evidence exists for the effectiveness of various types of professional self-management support interventions for informal caregivers of persons with dementia?

- The secondary question is:
  Which participant and intervention characteristics of self-management support interventions for informal caregivers of people with dementia are associated with larger effects?

## Methods

We conducted a meta-review, in the sense of a systematic review of systematic reviews, following, for as much as possible and applicable for this type of study, the Preferred Reporting Items for Systematic Reviews and Meta-Analyses (PRISMA) Statement [7] (see Additional file 1).

### Eligibility criteria

#### Types of study

Only systematic reviews were included. We considered a review to be systematic if the following criteria were met: (a) search terms must be described and (b) a search was conducted in PubMed and at least one other international scientific database. References were excluded if no effect studies (i.e. Randomized Controlled Trial (RCT), Controlled Clinical Trial (CCT) or quasi experimental designs) were included.

#### Types of participant

The systematic reviews to be included had to focus on informal caregivers of persons with dementia. No limitations concerning age were applied.

#### Types of intervention

The systematic reviews to be included had to focus on professional self-management support interventions. We considered an intervention, provided by the professional, to be a self-management support intervention if it explicitly focused on helping the informal caregiver to deal with the relative’s dementia and its consequences in everyday life. There must also have been direct or indirect (by phone/email) contact between the informal caregiver and the health care professional providing the intervention. Effects of self-management support interventions must be described and analyzed, and an overall conclusion must be drawn about the effectiveness of these interventions.

#### Types of outcome measure

Only systematic reviews presenting effects on informal caregivers of persons with dementia were included. References were excluded if the systematic reviews were primarily intended to address effects regarding health professionals or if they only described effects on the person with dementia.

### Search strategy and information sources

In March 2014, systematic literature searches were conducted in PubMed, CINAHL (Cumulative Index to Nursing and Allied Health Literature), Cochrane Library, Embase and PsycINFO to find relevant systematic reviews that met all the eligibility criteria. A sensitive search strategy was constructed first for PubMed/Medline, and subsequently adapted for the other databases used. The detailed search strategy for PubMed can be found in Additional file 2. All publications until March 2014 were taken into consideration. No language restrictions were imposed. References retrieved from the searches were entered into EndNote (version X7). After duplicates were eliminated, the selection of studies was carried out.

### Study selection

The protocol for study selection was as follows. References were identified for inclusion in two steps. First, the explicit pre-defined inclusion criteria described were applied to titles and abstracts of references identified from the search strategies. One reviewer (JGH) screened all references and the second reviewer (RV) independently checked a 10 % random selection of the references. If the level of agreement between the two reviewers was substantial to good (Kappa 0.60–0.80) [8] for the 10 % random selection, the first reviewer could proceed individually. If title and/or abstract provided insufficient information to assess the relevance, these references proceeded to the second inclusion stage. Second, full texts of the references selected in the first stage were independently screened by the reviewers. When the first and second reviewer did not agree on inclusion or exclusion, a third reviewer was consulted.

### Methodological assessment

After study selection, the methodological quality of the selected references was determined using the Quality Assessment Checklist for Reviews of Oxman and Guyatt [9]. Additional instructions by the authors of another meta-review [10], using the Quality Assessment Checklist for Reviews of Oxman and Guyatt, were applied to explicate the decisions for assessment (see Additional file 3). This checklist includes nine criteria for quality assessment of systematic reviews. The scientific quality is rated according to whether the review fulfilled, partially fulfilled or did not fulfill the following nine criteria by reporting or performing: (a) a search method, (b) a comprehensive search, (c) inclusion criteria, (d) selection bias, (e) validity of studies, (f) assessment criteria, (g) methods used to combine findings, (h) findings addressing the primary question of the review and (i) conclusions supported by data. Based on these nine criteria, the reviewer must give a rating score on a grading scale from one, reflecting extensive flaws, to seven, reflecting minimal flaws. The mean of the rating scores of the reviewers was calculated. If the scores differed by more than 1 point, the reviewers discussed their assessments and came to a new joint score. Reviews were considered to be of ‘high quality’ if the review was evaluated with a score between five and seven reflecting ‘minor flaws’ and ‘minimal flaws’ respectively. Only these high quality reviews were considered for inclusion since it is known that reviews judged as having critical flaws may be unsuitable for guiding health care decisions [11].

### Data-collection process

To investigate different aspects of the interventions, data were extracted from the systematic reviews. Data extraction was executed by the first reviewer (JGH) and subsequently checked by a second reviewer (BM). Extracted data included information about the study aim, search strategy described, databases used, target population, type of interventions, intended outcomes, design of the studies included, characteristics of the interventions, characteristics of implementation strategies, professionals’ characteristics, patient characteristics, environmental characteristics and overall conclusions.

### Data-analysis and synthesis

The underlying interventions in the included reviews were grouped based on the categorization of five targets described by Martin, et al. [6]. A self-management support intervention could have one or multiple intervention targets. Martin et al. describes the following self-management targets:

- *Relationship with family/friends/“carer”* focuses on the importance of the relationship and the challenges for both parties to ensure it is supportive.
- *Maintaining an active lifestyle* addresses the perception that people with dementia should be encouraged to stay active or engage in meaningful or pleasurable activities.
- *Psychological wellbeing* focuses on improving or maintaining psychological wellbeing to improve quality of life but also to aid adjustment and alleviate the negative impact of low mood on cognitive processes.
- *Techniques to cope with memory change* involves tips and techniques for living with an impaired memory, to improve coping with memory loss.
- *Information* covers a wide range of topics including what dementia is as a disease, features of disease progression, what losses in functioning to expect, what medical and psychological interventions exist, resources such as financial benefits [6].
- *Multi-component interventions* consist of and integrate several of the aforementioned intervention targets of Martin, et al. [6].

Furthermore, an evidence synthesis was conducted to indicate the level of evidence for the effectiveness of self-management support interventions on a specific outcome. This synthesis takes into account the reported evidence in the reviews and the number of underlying studies included in the reviews on which that evidence is based. The criteria used to indicate the level of evidence were inspired by the review of Steultjens, et al. [12]. Since Steultjens, et al. [12] included only RCTs, we adapted the criteria for this meta-review. Table 1 shows that at least one high quality systematic review (based on at least two underlying effect studies) should report consistently positive significant effects on a specific outcome to establish evidence for a self-management support intervention.

**Table 1:** Principles of evidence synthesis of systematic reviews

*Evidence*
Consistent positive, significant effects on a specific outcome in at least one high quality systematic review (based on at least two underlying effect studies)
*Limited evidence*
Effects on a specific outcome in at least one high quality systematic review (based on one underlying effect study)
*Inconclusive evidence*
Inconsistent effects on a specific outcome, because at least one high quality systematic review (including at least two underlying studies) shows positive, significant effects, while other review(s) included did not find such effects.
*No evidence*
None of the included reviews found consistent positive, significant effects on a specific outcome.
*No research found*
None of the included reviews examined effects on a specific outcome.

## Results

### Study selection

**Fig. 1:**
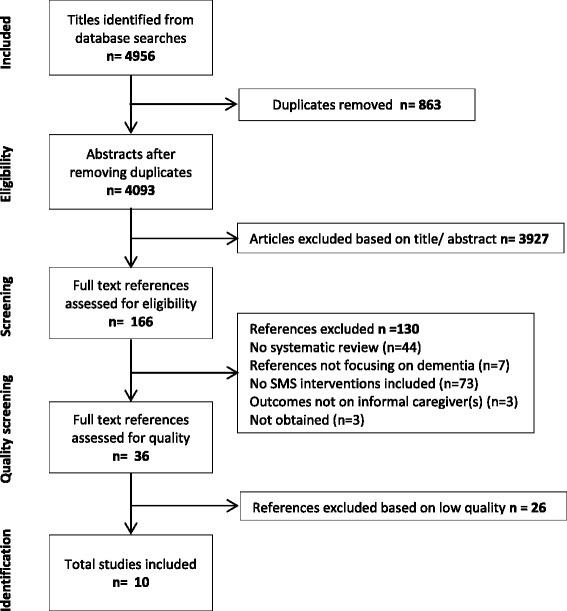
Flowchart study selection flow for systematic reviews

Four thousand nine hundred fifty-six references were identified from database searches. After merging the search results, all references were entered into EndNote, and 4093 references remained after duplicates were discarded. The first (JGH) and second reviewer (RV) reached substantial agreement on the 10 % random selection of the references (kappa coefficient of 0.71) [8] and therefore the remaining 90 % of the references were checked by the first author (JGH). 166 references remained after selection based on title and abstract. Full texts were searched for the 166 references, of which 163 were actually obtained. Four reviewers independently screened the full texts; the first reviewer (JGH) screened all full texts articles, one reviewer (PM) half of the total number of full texts, and two reviewers (RV, ALF) a quarter of the total full texts. Disagreement was resolved by discussion until consensus was reached. In total, 36 references remained after full text screening and were selected for the next stage of the review—the methodological assessment. Reasons for exclusion of the 130 references are detailed in Additional file 4. Three reviewers independently determined the methodological quality of the 36 references, of whom the first reviewer (JGH) screened all reviews and two reviewers (ALF, RV) both performed selection on half of the total number of included reviews. Ten reviews were evaluated with a high quality score. These high scores were based on well-documented methodology and the assessment of validity of the included primary studies. Twenty-six reviews received a score between one and four, reflecting ‘extensive’ to ‘major’ flaws in respect of the checklist. The main reason for excluding these reviews was that they either did not take measures or did not report on measures to prevent selection bias. In conclusion, ten reviews were selected for data-extraction (see Fig. 1).

### Characteristics of the reviews included

Additional file 5 shows general and methodological characteristics of the ten reviews included.

#### Publication date, origin of authors, journals and design of included reviews

Publication dates of the reviews included ranged from 2003 to 2013. The majority were published in the past 5 years. Three of the reviews were conducted in the Netherlands [13–15], and the remainder in Australia [16], Brazil [17], Canada [18], Germany [19], Taiwan [20] and the United Kingdom [21], while a review written in German had a correspondence address in Italy [22]. All reviews had a systematic review design, and five also contained a meta-analysis [16, 17, 19–21].

#### Objectives of reviews included

All included reviews aimed to focus on the effectiveness of interventions. Half of the reviews focused on a specific type of intervention, e.g. internet-based interventions, support group interventions or case management interventions. Five reviews did not specify the type of intervention in advance and discussed a broader range of interventions. None of the included reviews explicitly used a definition of self-management or self-management support.

#### Eligibility criteria of reviews included

The target population in all reviews comprised informal caregivers of persons with dementia. The underlying studies in the reviews mainly evaluated the effectiveness of interventions (RCT, CCT, quasi-experimental design). Additional to the inclusion of these designs, two reviews also included systematic reviews [16, 22]. Reported outcomes of the intervention varied in the reviews; see Additional file 6. Restrictions in the reviews were mainly related to language: six reviews reported language restrictions [13, 14, 16, 19, 20, 22]; two reviews explicitly reported to have no language restrictions [17, 21].

#### Information sources and search periods of the reviews included

All included reviews performed a comprehensive search in at least three databases [range 3 to 15]. PubMed was used in all reviews and the Cochrane Library in most. In addition, almost all reviews carried out other searches such as manual searches or searches of references listed in the reviewed studies. In seven reviews [13, 15, 17–21], the search comprised an extensive publication period of 10 years or more; three reviews had a shorter search period [14, 16, 22]. However, two of these concerned an update of an earlier review and thus included earlier reviews or the related underlying studies.

#### Score of methodological assessment of the reviews included

Six reviews received a quality score of 5.0 or 5.5, reflecting ‘minor flaws’ [13, 14, 16, 18, 21, 22]. Four reviews were found to have ‘minimal flaws’ based on quality score of 6.0 or 7.0 [15, 17, 19, 20]. Three reviews [15, 17, 20] received a quality score of 7.0 indicating that they met all quality requirements of the Quality Assessment Checklist for Reviews.

#### Number, design and control conditions of underlying studies in the reviews included

In total, 313 underlying studies were included in the reviews (range 7–127). In these underlying studies, 292 interventions are considered to be self-management support interventions based on the inclusion criteria of this meta-review. Generally, almost all reviews included only RCTs. The control conditions mainly involved usual care or a limited version of the intervention.

#### Number of intervention sessions, intervention period and professional who delivered the intervention

The number of intervention sessions and/or intervention periods were often not described by the included reviews. Some reviews reported these characteristics for a number of the underlying studies; accordingly, a range for intervention sessions and intervention periods is given. Few studies contained information on the professional who delivered the intervention. In those reviews that contained this information, nurses and case managers were the most frequently reported professionals [see Additional file 5].

### Results of underlying studies in reviews included

The underlying self-management support interventions of the included reviews and their reported outcome measures differed too much for their results to be pooled. Therefore, the interventions and results are categorized on the basis of the targets distinguished by Martin, et al. [6]: *relationship with family/friends/“carer”, maintaining an active lifestyle, psychological well-being, techniques to cope with memory change, information* and *multi-component interventions*. Within each category, first the different types of self-management support interventions are presented and the overall goal of each self-management support intervention is stated. Second, evidence for the self-management support interventions is presented based on the outcome. Additional file 6 presents the outcomes and effects of the reviews included. The most reported outcomes in the included reviews and the reported effectiveness of the self-management support interventions are shown in Additional file 7.

#### Self-management support interventions targeting relationship with family/friends/“carer”

Four reviews [16, 18, 19, 22] described self-management support interventions which target a supportive *relationship* between the person with dementia and the informal caregiver. Three reviews [18, 19, 22] described case management interventions; one review [16] included support interventions involving care planning and case management; and one review [18] described psychotherapy interventions.

Case management interventions under this target included advice and support by a health professional aimed at resolving personal problems that complicate informal care giving, to reduce conflict between caregivers and care recipients, and to improve family functioning.

Support interventions under this target consisted of supporting caregivers in their role involving care planning and case management.

Psychotherapy interventions consisted of individual and family counseling that focused on communication and problem-solving in relation to caregiving.

Using the described method for evidence synthesis, inconclusive evidence exists for the effectiveness of self-management support interventions, that focus on family relationships, for relieving caregiver burden [16, 19, 22] and enhancing coping skills [22]. All reviews that reported on caregiver depression presented no evidence [18, 19]. Other outcomes for which no evidence was found for the caregiver included subjective wellbeing and ability/knowledge [19]. None of the included reviews examined effects on self-efficacy, decision-making confidence, anxiety, stress, Revised Memory and Behavior Problem Checklist (RMBPC), quality of life, mood, health and sense of competence.

#### Self-management support interventions targeting the maintenance of an active lifestyle

None of the included reviews described self-management support interventions targeting the *maintenance of an active lifestyle* with effects on the informal caregiver.

#### Self-management support interventions targeting psychological wellbeing

Four of the reviews included described self-management support interventions targeting *psychological wellbeing* [15, 19, 20, 22]. In this category different types of interventions are categorized including caregiver support group interventions [20], psychotherapeutic interventions, support interventions [22], cognitive behavioral therapy, general support [19] and cognitive reframing interventions [15].

Support interventions, i.e. caregiver support groups and general support, under this target consisted of mutual emotional support for informal caregivers where they can share personal feelings, experiences and knowledge with other informal caregivers in order to relieve the pressure and burden of caregiving.

Therapeutic interventions, i.e. psychotherapy and cognitive behavior therapy, under this target involve dealing with difficult care situations and caregiving demands, and fostering activities that may promote subjective well-being.

Cognitive reframing interventions “focus on changing self-defeating or distressing cognitions into those cognitions that support adaptive behavior and reduce anxiety, depression and stress” [15].

Synthesizing these interventions under this target, evidence was found for self-management support interventions targeting psychological wellbeing for relieving stress or distress [15] and positive social outcomes [20]. Inconclusive evidence was found for the effectiveness of self-management support interventions targeting psychological wellbeing on relieving burden [15, 19, 20, 22], reduced depressive symptoms [15, 19, 20, 22], improving caregiver wellbeing [19, 20] and alleviating anxiety [15, 22].

No evidence was found for ability/knowledge [19], coping skills, self-efficacy and RMBPC [15]. None of the included reviews examined effects on the following outcomes reported in the included reviews: decision-making confidence, quality of life, mood, health and sense of competence.

#### Self-management support interventions targeting techniques to cope with memory change

Two of the reviews included described self-management support interventions targeting *techniques to cope with memory change* [19, 22].

Training programs under this target consisted of skills training for the informal caregivers, for example, to improve communication and problem solving skills. The person with dementia may possibly also be involved in the program, for example, in cognitive stimulation, ADL training and physical activity. Because physical and cognitive decline and behavior problems in the care recipient are associated with caregiver burden and depression, memory clinics and programs aimed at improving the competence of the care recipient may also have a positive effect on caregiver outcomes.

Limited evidence was found for the outcomes coping skills, mood and competence of the informal caregiver [22]. Inconclusive evidence was found for caregiver burden. No evidence was found for the effects of self-management support interventions targeting *techniques to cope with memory* change on caregiver depression, subjective wellbeing and ability/knowledge [19, 22]. None of the included reviews examined effects on self-efficacy, decision-making confidence, anxiety, stress/distress, RMBPC, quality of life, social outcomes and health.

#### Self-management support interventions targeting information

Seven of the reviews included described self-management support interventions targeting *information* [13, 16–19, 21, 22]. In this category, different types of interventions were categorized including (psycho-) educational interventions [16–19, 22], internet-based interventions [13], computer-networking interventions [18] and information and support interventions [21].

(Psycho-) Educational interventions under this target consisted of providing interdisciplinary education and knowledge about dementia, and teaching (coping) skills to support caregivers in their role. Pinquart and Sorensen [19] add that support may constitute part of psycho education, but is secondary to the educational content [19].

Internet-based computer-networking interventions under this target comprised education provision, decision-making support, communication and an opportunity for questions and answers for informal caregivers (through a computer network).

Evidence was found for the effectiveness of interventions targeting information on ability/knowledge [19, 22]. Limited evidence was found for caregiver stress [13], decision-making confidence [13, 18] and sense of competence [13]. One underlying study found a reverse effect on the outcomes anxiety, depression, well-being and quality of life. Anxiety and depression decreased significantly and well-being and quality of life increased in the control group whereas people in the online intervention group did not improve with respect to these outcomes [13]. Inconclusive evidence was found for improving caregiver burden, depression, well-being and self-efficacy [13, 16, 19, 22]. For coping skills and quality of life, two underlying studies had inconclusive findings. No evidence was found for caregiver health [16]. No research was found addressing RMBPC, social outcomes and mood.

#### Multi-component interventions

Four reviews [14, 16, 19, 22] included multi-component interventions. Multi-component interventions under this target consisted of a combination of various forms of interventions such as information, (psycho) education, support skills training and coping strategies for the caregiver and may also involve training for activities of daily life (ADL), walking or exercise and environmental adaptations for the person with dementia.

Inconclusive evidence was found for the effectiveness of multi-component interventions on caregiver burden, depression, quality of life, mood and sense of competence [14, 16, 19, 22]. No evidence was found for well-being and ability/knowledge [14, 16, 19, 22]. None of the included reviews examined effects on coping skills, self-efficacy, decision-making confidence, anxiety, stress/distress, RMBPC, social outcomes and health.

### Intervention and participant characteristics

Two reviews additionally performed analyses on intervention and participant characteristics [19, 20]. The review of Chien, et al. [20] conducted subgroup and regression analyses on intervention and participant characteristics, and their association with outcomes. Associations between these characteristics and effects were found in this review for the following characteristics: (psycho) educational groups, use of theoretical models, group size (6–10 people), group course (≥8 weeks) and intensity (≥16 h), follow up, leader background (interdisciplinary), female participation and age [20].

The review of Pinquart and Sorensen [19] also analyzed the association between intervention and participant characteristics. Associations for some outcomes were found for longer interventions (number of sessions, not further specified) and higher percentage of women [19].

## Discussion

This meta-review shows that scientific evidence exists for professional self-management support interventions targeting *psychological wellbeing* of informal caregivers of people with dementia. Effective interventions within this target were caregiver support group interventions, which were shown to relieve stress [15]; and cognitive reframing interventions that were shown to improve caregivers’ social outcomes such as social support, relationship with the patient and life quality [20]. Evidence was also found for the effectiveness of professional self-management support interventions targeting *information* on increasing caregivers’ knowledge. Examples of effective interventions in this target are psycho-educational interventions [19, 22].

Limited evidence was found for the effectiveness of self-management support interventions targeting *techniques to cope with memory change* on improving coping skills, mood and competence of informal caregivers [22]. Training programs are examples of these self-management support interventions. Further, limited evidence was also found for some interventions targeting *information* on improving decision-making confidence, stress and sense of competence [13, 18]. Examples for these interventions are internet-based support interventions and computer-networking interventions.

Inconclusive evidence was found for self-management support interventions targeting *relationship with family* and targeting *techniques to cope with memory change* on relieving caregiver burden. Self-management support interventions targeting *psychological wellbeing* were also found to have inconclusive findings on four caregiver outcomes including: burden, depression, wellbeing and anxiety. In the self-management support target *information,* inconclusive evidence was found on relieving burden and depression or improving wellbeing and self-efficacy in the informal caregiver. For multi-component interventions, inconclusive evidence was found on caregiver burden, depression, quality of life, mood and sense of competence.

Not much research was found on the informal caregiver outcomes self-efficacy, decision-making confidence, anxiety, stress or distress, RMBPC, quality of life, social outcomes, mood, health and sense of competence. Besides, none of the included reviews described effects of self-management support interventions targeting *maintaining an active lifestyle.*

We also aimed to identify specific intervention or participant characteristics that contributed to the effectiveness of these interventions. Two systematic reviews additionally performed analyses to investigate this. It is notable that both reviews found that in particular group course (≥8 weeks) and intensity (≥16 h) and longer interventions (number of sessions, not further specified) are associated with larger effects [19, 20]. These findings are in line with previous reviews, which also describe the importance for longer interventions or follow-up [23, 24].

The reviews in this meta-review studied different types of self-management support interventions. There was a considerable amount of variability between the underlying studies regarding, for example, content of the intervention, measurement tools used and implementation of the intervention. Despite this variability, it is noteworthy that psycho-education was integrated in most self-management support interventions that were found to be effective. For example, effective caregiver support group interventions consisted in most cases of a (psycho) educational group. Furthermore, it was shown that psycho-educational groups had a significantly higher effect on the outcome variables psychological well-being and depression [20]. The review of Pinquart and Sorensen [19] analyzed psycho-educational interventions with active participation of caregivers versus psycho-educational interventions that only provided information. Both interventions increased caregivers’ knowledge, but psycho-educational interventions with active participation of the caregiver had the broadest effects. An example of a psycho-educational intervention included in the review of Pinquart and Sorensen [19] was a intervention described by Hébert, et al. [25]. In this study, a group-intervention was tested consisting of fifteen two-hour weekly sessions and contained two components (cognitive appraisal and coping strategies). The intervention was aimed at primary caregivers of community-dwelling persons with dementia [25].

Looking at the main outcomes of the meta-review, the self-management support target of the successful interventions was directly related to the outcomes in informal caregivers. For example, self-management support interventions targeting *information* were found to be effective for improving the ability/knowledge of informal caregivers. This could also explain why we found no evidence for the effectiveness of interventions in the targets *maintaining an active lifestyle* on informal caregiver outcomes, since they were more focused on persons with dementia rather than on the informal caregiver.

### Implications for research and practice

Evidence exists for self-management support interventions targeting *psychological wellbeing* and *information* on specific caregiver outcomes; however more research is needed. To date, only limited research has been described in existent systematic reviews on, for example, the effect of self-management support interventions on quality of life or self-efficacy of the informal caregiver. This is remarkable because in many other studies on supporting self-management for people with long term conditions, it has been shown that self-management support can impact on these outcomes and that they are associated with each other [26]. Future research could focus on these outcomes for self-management support interventions for informal caregivers of persons with dementia.

Furthermore, more research is needed to investigate how effective interventions can be deployed and implemented. Although further investigation is needed, e-health was shown to be a promising extension to the currently offered care as usual [13]. Further research could take forward how self-management support interventions could be delivered by e-health.

Although self-evident, the results of this meta-review shows that it is important that the self-management support target is related to the main self-management need of the informal caregiver. For example, if health care providers want to improve caregivers’ social outcomes, they should focus on interventions targeting *psychological well-being*. Therefore it seems more beneficial to tailor a self-management intervention to the needs of the informal caregiver by using interventions that target on these specific needs.

This meta-review also indicated that longer interventions were associated with greater effects [19, 20] on some caregiver outcomes. This finding suggests that self-management support interventions should be given over an extended period of time and with a certain intensity.

Another relevant finding of this meta-review is that most of the effective self-management support interventions involved psycho-education. We therefore recommend health care professionals to consider psycho-education when focusing on self-management support targets *information* and *psychological well-being*.

### Strengths and limitations

To our knowledge, this is the first meta-review that gives insight into the level of evidence for the effectiveness of different types of self-management support interventions for informal caregivers of persons with dementia. Another important strength is the high methodological quality of the included reviews, indicating good reliability of the results which therefore may be appropriate for use in decision making [11].

Nonetheless, some limitations should be addressed. As mentioned earlier, none of the retrieved reviews labeled the interventions studies as ‘self-management support interventions’. Therefore, our selection of the reviews for inclusion and allocation of the reviews to intervention targets could contain an element of subjective judgment. An explicit definition of ‘self-management support’ interventions was used by the reviewers in order to minimize this.

Another limitation concerns the heterogeneity of the self-management support interventions within specific intervention targets regarding, for example, the nature and intensity of the interventions. This should be taken into account when interpreting the results.

Furthermore, a limitation is that the reported evidence in the reviews is sometimes partially based on the same underlying intervention reviews. An example of this is the review of Mantovan, et al. [22] that included, in addition to effect studies, systematic reviews (e.g. Thompson, et al. [21] and Pinquart and Sorensen [19]). However, the fact that Mantovan, et al. [22] included these two reviews did not change the conclusions of this meta-review.

## Conclusions

Evidence exists for professional self-management support interventions targeting *psychological wellbeing* and *information*. Health care professionals could take into account that psycho-education was integrated in most of the self-management support interventions that were found to be effective. Furthermore, longer and more intensive interventions were associated with higher effects.

## Additional files

Additional file 1:
**PRISMA 2009 checklist.** (DOC 74 kb) Additional file 2:
**Detailed search strategy Pubmed.** (PDF 58 kb) Additional file 3:
**Quality Assessment Checklist for Reviews and additional instructions by Francke et al. [** 10 **].** (PDF 71 kb) Additional file 4:
**Reason for exclusion of the excluded studies.** (PDF 84 kb) Additional file 5:
**General and methodological characteristics of reviews included.** (PDF 151 kb) Additional file 6:
**Outcome and effects of reviews included.** (PDF 140 kb) Additional file 7:
**Evidence for self-management support interventions reported by the included reviews on outcome.** (PDF 116 kb)
