# Supplementary material for: The effectiveness of interventions in supporting self-management of informal caregivers of people with dementia; a systematic meta review
Source: BMC Geriatr. 2015 Nov 11;15:147. doi: 10.1186/s12877-015-0145-6 (PMC4642777; doi:10.1186/s12877-015-0145-6)
Supplement: Additional file 2: — Detailed search strategy Pubmed. (PDF 58 kb) [file 12877_2015_145_MOESM2_ESM.pdf]

Additional file 2. Detailed search strategy PubMed

(Dementia[MeSH Terms] OR Cerebral Autosomal Recessive Arteriopathy with Subcortical Infarcts and Leukoencephalopathy [Supplementary Concept] OR Korsakoff Syndrome[Mesh] OR dement*[tiab] OR alzheim*[tiab] OR binswanger*[tiab] OR CADASIL[tiab] OR CARASIL[tiab] OR cjd[tiab] OR Creutzfeld Jacob[tiab] OR Creutzfeld Jakob[tiab] OR Creutzfeldt Jacob[tiab] OR Creutzfeldt Jakob[tiab] OR Frontotemporal Degenerat*[tiab] OR hiv associated neurocognitive disorder*[tiab] OR Huntington*[tiab] OR Kluver-Bucy*[tiab] OR Korsakoff*[tiab] OR Lewy Body[tiab] OR Pick Disease*[tiab] OR Picks Disease*[tiab] OR Pick's Disease*[tiab] OR Primary Progressive Aphasia*[tiab] OR sundown syndrome[tiab] OR sundowning[tiab])

(self care[MeSH Terms] OR self efficacy[MeSH Terms] OR patient education as topic[MeSH Terms] OR caregiver[MeSH Terms] OR self care*[tiab] OR selfcare*[tiab] OR self mana*[tiab] OR selfmana*[tiab] OR self help*[tiab] OR selfhelp*[tiab] OR self efficacy*[tiab] OR selfefficacy*[tiab] OR patient educat*[tiab] OR supportive care*[tiab] OR carer*[tiab] OR caregiv*[tiab] OR chronic care model[tiab])

((((systematic review[ti] OR meta-analysis[pt] OR meta-analysis[ti] OR systematic literature review[ti] OR (systematic review[tiab] AND review[pt]) OR consensus development conference[pt] OR practice guideline[pt] OR cochrane database syst rev[ta] OR acp journal club[ta] OR health technol assess[ta] OR evid rep technol assess summ[ta] OR drug class reviews[ti]) OR (clinical guideline[tw] AND management[tw]) OR ((evidence based[ti] OR evidence-based medicine[mh] OR best practice*[ti] OR evidence synthesis[tiab]) AND (review[pt] OR diseases category[mh] OR behavior and behavior mechanisms[mh] OR therapeutics[mh] OR evaluation studies[pt] OR validation studies[pt] OR guideline[pt] OR pmcbook)) OR ((systematic[tw] OR systematically[tw] OR critical[tiab] OR (study selection[tw]) OR (predetermined[tw] OR inclusion[tw] AND criteri*[tw]) OR exclusion criteri*[tw] OR main outcome measures[tw] OR standard of care[tw] OR standards of care[tw]) AND (survey[tiab] OR surveys[tiab] OR overview*[tw] OR review[tiab] OR reviews[tiab] OR search*[tw] OR handsearch[tw] OR analysis[tiab] OR critique[tiab] OR appraisal[tw] OR (reduction[tw] AND (risk[mh] OR risk[tw]) AND (death OR recurrence))) AND literature[tiab] OR articles[tiab] OR publications[tiab] OR publication[tiab] OR bibliography[tiab] OR bibliographies[tiab] OR published[tiab] OR unpublished[tw] OR citation[tw] OR citations[tw] OR database[tiab] OR internet[tiab] OR textbooks[tiab] OR references[tw] OR scales[tw] OR papers[tw] OR datasets[tw] OR trials[tiab] OR meta-analy*[tw] OR (clinical[tiab] AND studies[tiab]) OR treatment outcome[mh] OR treatment outcome[tw] OR pmcbook)) NOT (letter[pt] OR newspaper article[pt] OR comment[pt]))
((Meta-Analysis[ptyp] OR Review[ptyp] OR systematic[sb])))
