# Supplementary material for: The effectiveness of interventions in supporting self-management of informal caregivers of people with dementia; a systematic meta review
Source: BMC Geriatr. 2015 Nov 11;15:147. doi: 10.1186/s12877-015-0145-6 (PMC4642777; doi:10.1186/s12877-015-0145-6)
Supplement: Additional file 3: — Quality Assessment Checklist for Reviews and additional instructions by Francke et al. [ 10 ]. (PDF 71 kb) [file 12877_2015_145_MOESM3_ESM.pdf]

Additional file 3. Quality Assessment Checklist for Reviews and additional instructions by Francke et al. 2008

Annex

Annex 1 - Quality Assessment Checklist for Reviews

REF ID: _____
Reviewer: ___

1. **Were the search methods used to find evidence (primary studies) on the primary question(s) stated?**

☐**NO** ☐**PARTIALLY** ☐**YES**

Comments:

2. **Was the search for evidence reasonably comprehensive?**

☐**NO** ☐**PARTIALLY** ☐**YES**

Comments:

3. **Were the criteria used for deciding which studies to include in the review reported?**

☐**NO** ☐**PARTIALLY** ☐**YES**

Comments:

4. **Was bias in the selection of articles avoided?**

☐**NO** ☐**PARTIALLY** ☐**YES**

Comments:

5. **Were the criteria used for assessing the validity of the studies that were reviewed reported?**

☐**NO** ☐**PARTIALLY** ☐**YES**

Comments:

6. Was the validity of all of the studies referred to in the text assessed using appropriate criteria (either in selecting studies for inclusion or in analyzing the studies that are cited)?

☐NO ☐PARTIALLY ☐YES

Comments:

7. Were the methods used to combine the findings of the relevant studies (to reach a conclusion) reported?

☐NO ☐PARTIALLY ☐YES

Comments:

8. Were the findings of the relevant studies combined appropriately relative to the primary question the review addresses?

☐NO ☐PARTIALLY ☐YES

Comments:

9. Were the conclusions made by the author(s) supported by the data and/ or analysis reported in the review?

☐NO ☐PARTIALLY ☐YES

Comments:

10. Overall, how would you rate the scientific quality of this review?

Extensive flaws		Major flaws		Minor flaws		Minimal flaws
1	2	3	4	5	6	7

Comments:………………………

Instructions for use (from Oxman & Guyatt)

The purpose of this index is to evaluate the scientific quality (i.e. adherence to scientific principles) of research overviews (review articles) published in the medical literature. It is not intended to measure literary quality, importance, relevance, originality, or other attributes of overviews.

The index is for assessing overviews of primary ("original") research on pragmatic questions regarding causation, diagnosis, prognosis, therapy or prevention. A research overview is a

survey of research. The same principles that apply to epidemiologic surveys apply to overviews: a question should be clearly specified, a target population identified and accessed, appropriate information obtained from that population in an unbiased fashion, and conclusions derived, sometimes with the help of formal statistical analysis, as is done in "meta-analyses". The fundamental difference between overviews and epidemiologic surveys is the unit of analysis, not the scientific issues that the questions in this index address.
Since most published overviews do not include a methods section it is difficult to answer some of the questions in the index. Base your answers, as much as possible, on information provided in the overview. If the methods that were used are reported incompletely relative to a specific item, score that item as "partially". Similarly, if there is no information provided regarding what was done relative to a particular question, score it as "can't tell", unless these is information in the overview to suggest either that the criterion was or was not met.
For question 8, if no attempt has been made to combine findings, and no statement is made regarding the inappropriateness of combining findings, check "no". lf a summary (general) estimate is given anywhere in the abstract, the discussion or the summary section of the paper, and it is not reported how that estimate was derived, mark "no" even if there is a statement regarding the limitations of combining the findings of the studies reviewed. If in doubt mark "can't tell".
For an overview to be scored as "yes" on question 9, data (not just citations) should be reported that support the main conclusions regarding the primary question(s) that the overview addresses.
The score for question 10, the overall scientific quality, should be based on your answers to the first nine questions. The following guidelines can be used to assist with deriving a summary score: If the "can't tell" option is used one or more times on the preceding questions, a review is likely to have minor flaws at best and it is difficult to rule out major flaws (i.e. a score of 4 or lower) .If the "no" option is used on questions 2, 4, 6 or 8, the review is likely to have major flaws (i.e. a score of 3 or less, depending on the number and degree of the flaws).

Additional instructions by Francke et al. 2008:
In addition to the instructions of Oxman & Guyatt ("Index of the scientific quality of research overviews: instructions for use"), we made the following decisions for assessment:
- With regard to question 1 (search methods stated?) "partially" is filled in when only the search terms are presented. The score "yes" is given when it is also explained how these search terms are combined (by "AND", "OR" etc.).
- For question 2 (search reasonably comprehensive?) "partially" is marked in when only Pubmed/Medline has been searched or when the search period is shorter than 10 years.
- Regarding question 3 (criteria for inclusion) "yes" is scored when the inclusion criteria are explicitly described. When only exclusion criteria are presented, the score "partially" is given.
- With regard to question 4 (bias in selection avoided?) the score "yes" is given when both the initial selection based on references (titles and/or abstracts) and the subsequent selections (based on full texts) are performed by two independently working reviewers. When only a part of the selection steps is completed by two reviewers independently, then "partially" is filled in.

- For question 5 (criteria used for assessing the validity of the reviewed studies reported?) the score “yes” is marked when the reviewer(s) used explicit assessment criteria or an existing assessment checklist.
- With regard to question 6 (validity of all studies assessed using appropriate criteria; either in selecting or in analysing studies?) “yes” is filled in when both in the selection phase and during analysis the methodological quality of the studies is taken into account using explicit criteria. When only the rigorousness of research designs is taken into account, the score “partially” is marked.
- For question 7 (methods used to combine findings reported?) “yes” is scored when a “best evidence synthesis” is performed, when pooling of results has taken place or when results are presented on the basis of an “evidence table”.
- Regarding questions 8, 9 and 10, see instructions of Oxman & Guyatt.
