# Supplementary material for: The effectiveness of interventions in supporting self-management of informal caregivers of people with dementia; a systematic meta review
Source: BMC Geriatr. 2015 Nov 11;15:147. doi: 10.1186/s12877-015-0145-6 (PMC4642777; doi:10.1186/s12877-015-0145-6)
Supplement: Additional file 4: — Reason for exclusion of the excluded studies. (PDF 84 kb) [file 12877_2015_145_MOESM4_ESM.pdf]

Additional file 4. Reason for exclusion of the excluded studies

Study	Reason for exclusion
Annerstedt 2011	Excluded based on criteria ‘type of participant’
Anonymous 2013	Excluded based on criteria ‘type of study’
Arbesman 2011	Excluded based on criteria ‘type of study’
Archer 2014	Excluded based on criteria ‘type of study’
Aung 2010	Excluded based on criteria ‘type of study’
Aung 2012	Excluded based on criteria ‘type of study’
Ayalon 2006	Excluded based on criteria ‘type of intervention’
Bahar-Fuchs 2013	Excluded based on criteria ‘type of intervention’
Baldwin 2010	Excluded based on criteria ‘type of study’
Bharucha 2009	Excluded based on criteria ‘type of intervention’
Biem 2003	Excluded based on criteria ‘type of intervention’
Brodaty 2012	Excluded based on criteria ‘type of intervention’
Brody 2013	Excluded based on criteria ‘type of participant’
Buettner 2010	Excluded based on criteria ‘type of intervention’
Caddell 2011	Excluded based on criteria ‘type of intervention’
Campbell 2012	Excluded based on criteria ‘type of intervention’
Carrion 2013	Excluded based on criteria ‘type of intervention’
Carswell 2009	Excluded based on criteria ‘type of intervention’
Cepoiu-Martin 2012	Excluded based on criteria ‘type of study’
Chatterton 2010	Excluded based on criteria ‘type of intervention’
Christofoletti 2007	Excluded based on criteria ‘type of study’
Clare 2003	Excluded based on criteria ‘type of intervention’
Cohen-Mansfield 2001	Excluded based on criteria ‘type of study’
Cooke 2001	Excluded based on criteria ‘type of intervention’
Coon 2009	Excluded based on criteria ‘type of study’
Cooper 2007	Excluded based on criteria ‘type of intervention’
Courtenay 2010	Excluded based on criteria ‘type of participant’
Croot 2009	Excluded based on criteria ‘type of study’
Cummings 2009	Excluded based on criteria ‘type of study’
Daly 1999	Excluded based on criteria ‘type of study’
DePalma 2007	Excluded based on criteria ‘type of study’
Doody 2001	Excluded based on criteria ‘type of intervention’
Drennan 2012	Excluded based on criteria ‘type of intervention’
Egan 2010	Excluded based on criteria ‘type of intervention’
Egan 2006.	Excluded based on criteria ‘type of study’
Eggenberger 2013	Excluded based on criteria ‘type of study’
Eggermont 2006	Excluded based on criteria ‘type of intervention’
Enmarker 2011	Excluded based on criteria ‘type of intervention’
Etters 2008	Excluded based on criteria ‘type of study’
Eustice 2000	Excluded based on criteria ‘type of participant’
Filan 2006	Excluded based on criteria ‘type of intervention’

Fischer-Terworth 2009	Excluded based on criteria ‘type of intervention’
Forbes 2008	Excluded based on criteria ‘type of intervention’
Forbes 2013	Excluded based on criteria ‘type of intervention’
Forbes 1998	Excluded based on criteria ‘type of intervention’
Gallagher 2011	Excluded based on criteria ‘type of study’
Gillespie Lesley 2012	Excluded based on criteria ‘type of intervention’
Gitlin 2012	Excluded based on criteria ‘type of study’
Gonzalez-Guillermo 2008	Excluded based on criteria ‘type of study’
Hall 2012	Excluded based on criteria ‘type of intervention’
Hautzinger 2002	Excluded based on criteria ‘type of study’
Herrmann 2007	Excluded based on criteria ‘type of intervention’
Hermann 2008	Excluded based on criteria ‘type of intervention’
Herrmann 2001	Excluded based on criteria ‘type of intervention’
Hodgkinson 2007	Excluded based on criteria ‘type of intervention’
Hort 2010	Excluded based on criteria ‘type of study’
Hulme 2010	Excluded based on criteria ‘type of study’
Innes 2011	Excluded based on criteria ‘type of intervention’
Jones 2012	Excluded based on criteria ‘type of intervention’
Kiepe 2012	Excluded based on criteria ‘type of study’
Kim 2012	Excluded based on criteria ‘type of intervention’
Koger 2000	Unobtainable
Kong 2009	Excluded based on criteria ‘type of intervention’
Konno 2013	Excluded based on criteria ‘type of intervention’
Kotronoulas 2013	Excluded based on criteria ‘type of intervention’
Kuske 2007	Excluded based on criteria ‘type of intervention’
Kverno. 2009	Excluded based on criteria ‘type of intervention’
Lawrence 2012	Excluded based on criteria ‘type of intervention’
LeClerc 1998	Excluded based on criteria ‘type of study’
Livingston 2005	Excluded based on criteria ‘type of study’
Lopez 2007	Excluded based on criteria ‘type of intervention’
Lorenz 2008	Excluded based on criteria ‘type of intervention’
Manckoundia 2008	Excluded based on criteria ‘type of study’
Marcus 1992	Excluded based on criteria ‘type of study’
McKeown 2006	Excluded based on criteria ‘type of intervention’
McLaren 2013	Excluded based on criteria ‘type of study’
Moniz Cook 2012	Excluded based on criteria ‘type of intervention’
Napoles 2010	Excluded based on criteria ‘type of intervention’
Neal 2003	Excluded based on criteria ‘type of intervention’
Nguyen 2008	Excluded based on criteria ‘type of intervention’
Nijhof 2009	Excluded based on criteria ‘type of study’
O'Connor 2011	Excluded based on criteria ‘type of study’
O'Connor 2009	Excluded based on criteria ‘type of participant’
Olazaran 2010	Excluded based on criteria ‘type of intervention’
O'Neil 2011	Excluded based on criteria ‘type of intervention’
Opie 1999	Excluded based on criteria ‘type of intervention’

Orgeta 2014	Excluded based on criteria ‘type of intervention’
Orgeta 2014	Excluded based on criteria ‘type of outcome measure’
Padilla 2011	Excluded based on criteria ‘type of intervention’
Paolino 2013	Excluded based on criteria ‘type of study’
Parks 2006	Excluded based on criteria ‘type of study’
Penrose 2005	Excluded based on criteria ‘type of intervention’
Perkins 2008	Excluded based on criteria ‘type of intervention’
Pimouguet 2010	Excluded based on criteria ‘type of intervention’
Powell 2008	Excluded based on criteria ‘type of intervention’
Preschl 2011	Excluded based on criteria ‘type of participant’
Price 2000	Excluded based on criteria ‘type of intervention’
Rae 2011	Excluded based on criteria ‘type of study’
Rice 2001	Excluded based on criteria ‘type of study’
Rigaud 2011	Excluded based on criteria ‘type of study’
Roberts 2000	Excluded based on criteria ‘type of intervention’
Robinson 2006	Excluded based on criteria ‘type of outcome measure’
Robinson 2007	Excluded based on criteria ‘type of intervention’
Santos 2011	Excluded based on criteria ‘type of intervention’
Schneider 1993	Excluded based on criteria ‘type of study’
Schoenmakers 2010	Excluded based on criteria ‘type of intervention’
Schulz 2002	Excluded based on criteria ‘type of intervention’
Seitz 2012	Excluded based on criteria ‘type of intervention’
Selwood 2007	Excluded based on criteria ‘type of study’
Sorbi 2012	Excluded based on criteria ‘type of study’
Souder 2003	Excluded based on criteria ‘type of study’
Spijker 2008	Excluded based on criteria ‘type of outcome measure’
Spira 2006	Excluded based on criteria ‘type of study’
Taft 1995	Excluded based on criteria ‘type of intervention’
Tam-Tham 2013	Excluded based on criteria ‘type of intervention’
Teri 2005	Excluded based on criteria ‘type of intervention’
Thirymoorthy 2013	Unobtainable
Topo 2009	Excluded based on criteria ‘type of study’
Torti 2004	Excluded based on criteria ‘type of study’
Toseland 1989	Excluded based on criteria ‘type of participant’
Ueda 2013	Excluded based on criteria ‘type of intervention’
van Ginneken 2013	Excluded based on criteria ‘type of intervention’
Vazquez 2009	Excluded based on criteria ‘type of intervention’
Verkaik 2005	Excluded based on criteria ‘type of intervention’
Wall 2010	Excluded based on criteria ‘type of intervention’
Warner 2008	Unobtainable
Woods 2012	Excluded based on criteria ‘type of intervention’
Woodward 2013	Excluded based on criteria ‘type of study’
Yu 2009	Excluded based on criteria ‘type of intervention’

Zetteler 2008	Excluded based on criteria ‘type of intervention’
