# Supplementary material for: The effectiveness of interventions in supporting self-management of informal caregivers of people with dementia; a systematic meta review
Source: BMC Geriatr. 2015 Nov 11;15:147. doi: 10.1186/s12877-015-0145-6 (PMC4642777; doi:10.1186/s12877-015-0145-6)
Supplement: Additional file 5: — General and methodological characteristics of reviews included. (PDF 151 kb) [file 12877_2015_145_MOESM5_ESM.pdf]

Additional file 5. General and methodological characteristics of reviews included

General and methodological characteristics of reviews included						Underlying studies in the reviews		
**Reference, first author's country of origin**	**Review design**	**Review objective**	**Review's eligibility criteria**	**Information sources and search periods**	**Score methodological assessment:**	**Number, design and control conditions**	**Participants in underlying studies included in reviews**	**Number of sessions, intervention period and professional who delivered the intervention**
**Boots et al. (2013)**<br><br>Netherlands	Systematic review	(1) To present an overview of the evidence of the effectiveness and quality of Internet interventions for the informal caregivers of patients with dementia; (2) to assess which types of interventions are most effective (i.e., provide the best outcome for the participants); and (3) to indicate the feasibility of such interventions.	Inclusion criteria:<br>− studies that reported the effects (quantitative and qualitative) of an internet-based intervention<br>− informal (nonprofessional) caregivers of patients with mild cognitive impairment/dementia<br><br>Exclusion criteria:<br>− interventions solely aimed at the patient instead of the caregiver	PubMed, PsycINFO, CINAHL, Web of Science, and the Cochrane Library<br><br>Additional searches: searches of references listed in the reviewed papers and meta-analyses<br><br>Search period: 1988-2013	**5**	12 studies<br><br>Design: (pretest/posttest) RCT, mixed method, formative evaluation, quasi-experimental<br><br>Control conditions:<br>− usual care, waitlist controls<br>− a limited version of the intervention	Informal caregivers of persons with dementia. Setting was not further specified	Number of intervention sessions/modules ranged from 3 to 7<br><br>Intervention duration ranged from 4 weeks to 12 months<br><br>Professionals<br>− caregiver-therapist<br>− technical experts in caregiving
**Chien et al. (2011)**<br><br>Taiwan	Meta-analysis	To provide an integrated analysis of the effectiveness of support group intervention for caregivers of patients with dementia and to identify the significant predictors variables of outcomes	Inclusion criteria:<br>− nonprofessional caregivers of patients with dementia<br>− articles using quantitative methods and excluding qualitative studies<br>− support groups led by professionals<br>− support group types including mutual support groups, educational psychology groups, and educational training groups<br>− studies of quasi- or true experimental designs<br>− control groups were general care, waiting list, or minimal support, including caregiving manuals and videos, information guides, personal consultation, traditional support groups, and short-break services<br>− journal articles on caregivers' psychological well-being on burden and social consequence<br><br>Exclusion criteria:<br>− groups organized on the internet, by telephone, or in the community<br>− single-subject or single-group design	Cochrane, Medline, PubMed, PsycARTICLES, PsycINFO, ERIC, Ageline, CINAHL, Psychology and Behavioral Sciences Collection<br><br>Additional searches: Google search and reference lists of retrieved articles<br><br>Search period: 1998-2009	**7**	30 studies<br><br>Design of underlying studies is not reported (reported in inclusion criteria)<br><br>Control conditions:<br>− general care, waiting list<br>− minimal support	Spouse (27-100%) of persons with dementia. Setting was not further specified	Number of intervention sessions is not specified<br><br>Intervention duration (follow up after session) is not provided<br><br>Professionals<br>− single professional background<br>− interdisciplinary

**Mantovan et al. (2010)**<br><br>Italy (correspond ence address)	Systematic review	To collect the current state of knowledge regarding relief or promoting interventions for family caregivers of people with dementia from international studies	Inclusion criteria:<br>– caregiver and people with dementia (dyads) (> 18 years)<br>– the care takes place at home (home care) by caregiver<br>– relief and promotional offers through health and social service providers<br>–Outcomes: stress, depression, wellbeing / quality of life, coping skills / management, institutionalization<br>– quantitative studies of evidence Class 1-11 (Antes et al, 2003.)<br>– publications from 2004-2009<br>– English, German, and Italian language studies (English abstracts in foreign languages)<br><br>Exclusion criteria:<br>– frail children (<18 years)<br>– oncological care needed (terminal stage)<br>– nursing care with congenital disabilities<br>– care by professional nurses in social institutions<br>– pharmacological interventions<br>– studies of evidence Class 111-1V (Antes et al, 2003.)<br>– gray literature<br>– publications for 2004<br>– foreign language studies without English abstract	Cochrane Library, Medline (PubMed), Embase, Cinahl (Ebsco) und PsycINFO (Ebsco)<br><br>Additional searches: journals were searched for relevant studies and their references in English and German<br><br>Search period: 2004-2009	**5**	9 studies were reviewed<br><br>Design: RCT, CT, secondary data analyses. In addition, 3 systematic reviews and 2 systematic reviews with meta-analysis were included<br><br>Control conditions:<br>– a limited version of the intervention	Family caregivers (not further specified) of person with dementia living at home	Number of intervention sessions ranged from 5 to 6<br><br>Intervention duration ranged from 3 to 18 months<br><br>Professionals:<br>– case manager, volunteer
**Marim et al. (2013)**<br><br>Brazil	Systematic review and meta-analysis	To examine the evidence available in the literature on how effective education and support programs are in reducing the burden of caregivers of patients with dementia	Inclusion criteria:<br>– RCTs with blinded assessments<br>– interventions which provided interdisciplinary education and support for caregivers of patients with dementia<br>– outcomes: the scale of caregiver burden using the Zarit Burden Interview<br><br>Exclusion criteria:<br>– not provided	PubMed, LILACS, EMBASE, SciELO, The Cochrane Library, Web of Science, CINAHL<br><br>Additional searches: www.controledtrials.com database and abstracts of conference papers, references to review articles, published systematic reviews and references to randomized clinical trials<br><br>Search period: inception-2011	**7**	7 studies<br><br>All studies were RCTs<br><br>Control conditions:<br>– usual care	Caregivers (not further specified) of person with dementia. Setting was not further specified	Number of intervention sessions is not provided<br><br>Intervention duration ranged from 4 to 12 months<br><br>Professionals:<br>– not reported

**Parker et al. (2008)**<br><br>Australia	Systematic review and meta-analysis	To assess the effectiveness of interventions that assist caregivers to provide support for people living with dementia in the community	Inclusion criteria:<br>− caregivers who provide support for people with dementia living in the community<br>− three categories of intervention types:<br>(1) Interventions designed to support caregivers in their role (Skills training, Education to assist, Support groups/programs).<br>(2) Interventions of formal approaches to care designed to support caregivers in their role (Care planning, Case management, Specially designated members of the healthcare team (dementia nurse specialist or volunteers trained in caring for someone with dementia).<br>(3) Multi-component interventions that involve any of the above<br>− systematic reviews, meta-analyses, randomized control trials, quasi-experimental studies, cohort studies, case control studies and observational studies without control groups.<br>− studies from 2000 to 2005<br>− outcomes: Health service utilization, caregiver satisfaction with health service utilization, psychological morbidity of caregivers, caregiver quality of life, caregiver self-reported perception of knowledge or competence in caring for someone with dementia in the community<br><br>Exclusion criteria:<br>− outcomes concerning respite care	CINAHL, MEDLINE, PsycINFO, Cochrane (CDSR, DARE, CCTR, CENTRAAL), APAIS health, Current Contents, ERIC, Professional Development Collection, Psycarticles, Dissertation and Thesis Abstracts, NHMRC guidelines, Social Science Citation, Ageline, Econlit, Sociological Abstracts<br><br>Additional searches: hand searching reference lists of articles retrieved, foundation works older than five years referred to in the literature were also included in the review<br><br>Search period: 2000-2005	**5.5**	34 studies<br><br>All studies were RCTs. In addition 3 meta-analyses and 3 systematic reviews were included<br><br>Control conditions:<br>− usual care, waiting list<br>− a limited version of the intervention	Spouse (52%)<br>Caregivers taking responsibility for the care of a person with dementia living in the community	Number of intervention sessions ranged from 1 to 38<br><br>Intervention period:<br>post test ranged from 1 to 24 months<br>2nd post test ranged from 3 to 24 months<br>3rd post test ranged from 6 to 36 months<br><br>Professionals:<br>− project staff member, occupational therapist, psychologist, nurse(s), volunteers who had been caregivers
**Peacock & Forbes (2003)**<br><br>Canada	Systematic review	To determine the effectiveness of a range of interventions to enhance the well-being of caregivers of elderly persons with dementia living in the community	Inclusion criteria:<br>− studies published in 1992 or later<br>− interventions directed at caregivers of an elderly individual with dementia living in the community<br>− caregiver outcomes: well-being (physical, mental, social, or financial), depression, strain, and/or other (e.g., institutionalization, health-care expenditure)<br>− incorporated a control group or a pretest-posttest design with a sample size greater than one<br><br>Exclusion criteria:<br>− not provided	CINAHL, PubMed, PsycINFO<br><br>Additional searches: hand searching the table of contents of The Gerontologist, Journal of Gerontological Nursing, and Journal of the American Geriatrics Society as well as searches in the reference lists of retrieved studies<br><br>Search period: 1992-2002	**5.5**	11 studies<br><br>All studies were RCTs<br><br>Control conditions:<br>− usual care<br>− Interventions: respite care, a limited version of the intervention	Caregivers (not further specified) of persons with dementia living in the community	Number of interventions sessions ranged from 14 to 72<br><br>Intervention period ranged from 12 weeks to 8 years<br><br>Professionals:<br>family coordinator, case manager, clinical psychologist, clinical nurse specialist, family counselors, nurse

**Pinquart & Sörensen (2006)**<br><br>Germany	Meta-analysis	To investigate effects of interventions on caregiver burden, depression, indicators of positive subjective well-being, ability/knowledge of caregivers, symptoms of care receivers and the risk of institutionalization.<br><br>To compare effects of different forms of interventions (such as caregiver education, general support, and respite)<br><br>To analyze the impact of study characteristics on intervention effects, such as group-based versus individualized interventions	Inclusion criteria:<br>– care receiver has dementia<br>– an intervention condition was compared to a control condition that did not receive the treatment<br>– outcomes: caregiver burden, depression, subjective wellbeing (e.g. life-satisfaction, happiness), knowledge and/or coping abilities of the caregiver, CR symptoms and institutionalization<br>– statistics could be converted into effect sizes<br>– studies written in English or German, or in a language for which we could get translation<br><br>Exclusion criteria:<br>– not provided	PsycINFO, MEDLINE, Ageline, Psyndex<br><br>Additional searches: cross-referencing<br><br>Search period: 1982-2005	**6**	127 studies were reviewed of which 111 consist SMS interventions (16 studies are respite interventions)<br><br>Design of underlying studies are not reported<br><br>Control conditions were not reported	Spouse (60%), adults child (39%)<br>of people with dementia living at home (two exceptions)	Overall:<br>Number of intervention sessions ranged from 1 to 180 (median = 9).<br><br>Intervention period: range of intervention period is not provided. Follow up (in n=32) was on average after 11 months (SD=11.1)<br><br>Professionals:<br>– not reported
**Thompson et al. (2007)**<br><br>United Kingdom	Systematic review and meta-analysis	To assess the effectiveness of interventions based around information and support provision for informal caregivers of people with dementia in community settings	Inclusion criteria:<br>– RCTs<br>– principal informal caregiver (not a paid professional) and care recipient (diagnosed with dementia) dyad living in the community<br>– information and/or support interventions<br>– outcomes: caregiver outcomes (quality of life, physical and mental health, burden or satisfaction); and patient outcomes (activities of daily living or behaviours); health service utilization (numbers of in-patient, outpatient or primary health care contacts); economic outcomes (time spent on caring activities)<br><br>Exclusion criteria:<br>– not provided	Search of the Specialized Register of the Cochrane Dementia and Cognitive Improvement Group<br><br>Additional searches: citation searches for key papers, reference checking and contact with authors<br><br>Search period: inception - 2005	**5**	44 studies<br><br>All studies are RCTs<br><br>Control conditions:<br>– wait list, usual care<br>– interventions: a limited version of the intervention	Informal caregivers (individuals who provide extraordinary, uncompensated care, predominantly in the home, carried out primarily by family members) of persons with dementia living in the community	Number of sessions ranged from 2 to 72<br><br>Intervention period ranged from 1 month to 24 months<br><br>Professionals:<br>– nurse moderator, case manager, counselor, psychologist, volunteers

**Van 't Leven et al. (2013)**<br><br>Netherlands	Systematic review	To update Smits et al.'s (2007) systematic review, and to provide the current best evidence about psychosocial programs for the dyads that involve face-to-face contact between professional caregivers and both the patient and the caregiver	Inclusion criteria:<br>− effect studies evaluating dyadic psychosocial interventions for both older people with dementia living in the community and their caregivers.<br>− interventions had to involve face-to-face contact between care professional and the person with dementia as well as the informal caregiver and the same care professional<br>− interventions had to target psychosocial outcomes, improving mental health or well-being<br>− RCTs<br><br>Exclusion criteria:<br>− RCTs involving respite interventions, and technological devices, as well as cost-effectiveness studies, studies among nursing home residents, and integrated studies where results could not be related to a specific intervention or program	PsycINFO, EMBASE, MEDLINE, and CINAHL for single studies and reviews, and the Cochrane Library for systematic reviews<br><br>Search period: 2005-2012 (also update of review of Smits et al.)	**5.5**	23 studies<br><br>All studies were RCTs<br><br>Control conditions:<br>− usual care, waiting list<br>− interventions: educational materials/visits, medication<br>− not reported	Informal caregivers (not further specified) of person with dementia living in the community	Number of sessions ranged from 2 to 15<br><br>Intervention period ranged from 5 weeks to 3 years<br><br>Professionals:<br>− professional with master's degree, occupational therapist, multidisciplinary team, home health prof, nurse/case manager/coordinator, home care advisor psychiatrist, primary care physician/nurse practitioner/psychologists
**Vernooij-Dassen et al. (2011)**<br><br>Netherlands	Systematic review	To evaluate the effectiveness of cognitive reframing interventions for carers of people with dementia<br><br>To indicate the nature and quality of the evidence available<br><br>To contribute to providing an evidence base for clinical practice	Inclusion criteria:<br>− RCTs<br>− family carers taking care of a person with any type of dementia; spouse, child, other family member or friend<br>− interventions aimed at reduction of caregiver problems; family carers' beliefs about their responsibilities, about own need for support and assistance and their interpretations of behaviors of people with dementia. No restrictions regarding control interventions<br>− outcomes: psychological morbidity and distress of family carers (including depression and anxiety), QoL of family carers, family carers' appraisal of their role performance (burden, coping, self-efficacy and problem behaviors), healthcare utilization outcomes (admission to residential care or number of general practice visits)<br><br>Exclusion criteria:<br>− not provided	Cochrane Dementia and Cognitive Improvement Group (CDCIG) Specialized Register including The Cochrane Library, MEDLINE, EMBASE, PsycINFO, CINAHL and LILACS<br><br>Additional searches: ongoing trial databases and other gray literature sources<br><br>Search period: inception-2009	**7**	11 studies<br><br>All studies were RCTs<br><br>Control conditions:<br>− usual care, wait list<br>− interventions: telephone calls, multiple different control groups<br>− not reported	Spouse (40.2%)<br>Adult child (28.1%)<br>and other (6.7%)<br>of community-dwelling people with dementia<br><br>(25% of carers no relationship to person with dementia was specified)	Number of sessions ranged from 8 to 14<br><br>Intervention period ranged from 9 weeks to 4 months<br><br>Professionals:<br>− not reported
