# Supplementary material for: The effectiveness of interventions in supporting self-management of informal caregivers of people with dementia; a systematic meta review
Source: BMC Geriatr. 2015 Nov 11;15:147. doi: 10.1186/s12877-015-0145-6 (PMC4642777; doi:10.1186/s12877-015-0145-6)
Supplement: Additional file 6: — Outcome and effects of reviews included. (PDF 140 kb) [file 12877_2015_145_MOESM6_ESM.pdf]

Additional file 6. Outcome and effects of reviews included

Outcome and effects of reviews included					
**Reference**	**Type of self-management support interventions**	**Outcome variables of reviews included**	**Results of intervention as reported in review**	**Participant and intervention characteristics**	**Conclusion of the authors of included reviews**
**Boots et al. (2013)**	Internet-based interventions including:<br>− website with information and support<br>− website with additional caregiving strategies<br>− website combined with telephone support<br>− website with additional e-mail support<br>− website with a combination of individual work and exchange with other caregivers online	Various e.g.:<br>− self-efficacy<br>− stress/burden<br>− depressive symptoms<br>− coping<br>− social contact/ support<br>− knowledge<br>− (utilization of) health services	− (small) significant improvement of caregivers' well-being in measurement of depression, sense of competence, decision-making confidence, self-efficacy, and burden. However caregiver burden did not decrease significantly in all studies. Significant differences were found in caregiver burden between non-users and frequent users of the program. Increased intention to seek support was reported, as well as caregiver gain and self-control whereas caregiver stress and strain decreased<br>− no significant effects between groups or within the treatment group on quality of life or coping skills<br>− stress management techniques among caregivers and social isolation and health status did not improve. One study reported improvements in the control group: anxiety and depression decreased significantly, and well-being and quality of life increased.	No effects for participant and intervention characteristics were reported based on an analysis	Internet interventions for informal dementia caregivers may improve caregiver well-being
**Chien et al. (2011)**	Caregiver support group interventions including:<br>− mutual support group<br>− psychoeducational group<br>− educational group	− mental health<br>− depression<br>− burden<br>− social outcome	− caregivers' mental health significantly improved (g= -0.44[95% CI= -0.73, -0.15] p= <0.001)<br>− depression was significantly reduced in caregivers (g= -0.40[95%CI=-0.72,-0.08] p=<0.001)<br>− support group interventions effectively relieved burden for caregivers (g= -0.23 [95% CI= -0.33, -0.13] p=0.286)<br>− social outcomes significantly improved (g= 0.40 [95% CI=0.09, 0.71] p=<0.001)	− Intervention characteristics: use of theoretical models, length of group sessions and total hours impacted significantly on psychological well being and depression. The following factors were associated with a more prominent and larger effect size: psycho educational groups, use of theoretical models, group size of 6-10 people, length of group sessions ≥ 8 weeks and total hours ≥16 and follow up. For burden: educational and training group, use of theoretical models, group size of 6-10 people, length of group sessions ≥ 8 weeks and total hours ≥16 and follow up.<br><br>− Participant characteristics (female participants, younger age, severity of dementia) in between-groups comparison on the effect sizes of psychological well-being, depression and in some cases burden	Support groups are significantly effective for caregivers of patients with dementia, while the effect size varies with different outcome variables, including psychological well-being, depression, burden, and social outcomes

**Mantovan et al. (2010)**	− psycho-educational interventions<br>− psychotherapeutic interventions<br>− support interventions (individually or in groups)<br>− case and care management<br>− multicomponent interventions<br>− training programs	Various	− psycho-educational intervention: 1 study reported improvements in competence, coping and total burden. 1 underlying review (Thompson) reported that technology-based psycho educational interventions have no effect on depression. Group based interventions were found to have a positive effect on depression. Individual based interventions were found to have no effect on depression and self-efficacy. 1 underlying review (Pinquart) found significant effects for burden, depression, wellbeing and ability and knowledge<br>− psychotherapeutic interventions (cognitive-behavioral) lead to a clear improvement of depression, while this showed inconsistent results for other interventions.<br>− support interventions: 1 underlying review reported that none of their included studies showed effect on anxiety. 1 underlying study reported a reduction in depression. Another underlying study found no effects on burden and depression.<br>− counseling / case management: 1 underlying review (Pinquart) reported a reduction of burden. 1 underlying study reported increased coping and a determination of coping.<br>− multi-component: effects of underlying studies report significant effects in depression, burden and psychological health. In one underlying review (Pinquart) no effect was found for burden, depression, subjective wellbeing and ability/knowledge.<br>− training program: effects of underlying studies reported mixed results on effects on the caregiver. 1 underlying study reported a reduction in burden. One underlying review(Pinquart) reported no effects on the caregiver. Coping, mood and competence were also reported to be improved. A significant improvement of competence was only found after 6 months.	No effects for participant and intervention characteristics were reported based on an analysis	The presentation of the results shows that psycho-educational, relieving, supportive, psychotherapeutic and multi-component interventions offered as well as counseling and case/care management among caring family members have significant effects on parameters such as burdens, level of depression, subjective well-being, skills/knowledge.
**Marim et al. (2013)**	− (psycho) educational programs	− caregiver burden	− decreased caregiver burden (Articles that used intention to treat (MD -1.10 [95%CI-2.25, 0.05] p=0.06). Articles without intention to treat (MD:-4.46 [95%CI-15.54, 6.62] p=0.43). After excluding 3 studies (to reduce heterogeneity): MD -1.62 [95%CI -2.16, -1.08])	No effects for participant and intervention characteristics were reported based on an analysis	The evidence obtained in this study suggests that educational and support programs have a positive impact on the reduction of caregiver burden when compared to standard care
**Parker et al. (2008)**	− psychoeducational interventions<br>− support interventions<br>− multi-component interventions	Various including:<br>− depression<br>− health<br>− subjective well-being<br>− self-efficacy<br>− burden	− psycho-educational interventions: statistically significant results for depression (WMD -1.93[95%CI -3,79, -0.07] p=0.04) and subjective well-being (SMD -0.16 [95%CI -0.32, 0.00] p=0.04) but not for health (SMD 0.05 [95%CI -0.38, 0.48]), self-efficacy (SMD 0.30 [95%CI -0.04, 0.65]) and burden (SMD 0.02 [95%CI -0.37, 0.42])<br>− support interventions (2 studies out of 7) report significant effects on caregiver burden (SMD -0.41, [95% CI -0.80, -0.02], p=0.04). 5 studies did not report significant findings in their interventions<br>− multi-component: Mixed results were found in the underlying studies and reviews across a variety of outcome measures.	No effects for participant and intervention characteristics were reported based on an analysis	From this review there is evidence to suggest that well designed psycho-educational or multi-component interventions may assist caregivers of people with dementia who live in the community. More studies are needed to provide evidence of the effect of support interventions

**Peacock & Forbes (2003)**	− case-management interventions<br>− education interventions<br>− psychotherapy interventions<br>− computer-networking interventions	− caregiver depression<br>− caregiver strain<br>− caregiver stress<br>− use of formal services	− case management interventions did not impact on levels of strain or depression for caregivers.<br>− education interventions are insufficient to improve overall caregiver psychological well-being, such as decreasing strain and depression.<br>− psychotherapy: only outcomes on the person with dementia were given<br>− computer networking intervention: a significant increase in decision-making confidence; no significant differences between the groups were found in relation to decision-making skills, social isolation, or use of health services	No effects for participant and intervention characteristics were reported based on an analysis	This systematic review reveals few significant effects for caregiver interventions, although non-significant findings were more common.
**Pinquart & Sörensen (2006)**	Various interventions included namely:<br>− psycho-educational interventions<br>− cognitive behavioral therapy (CBT)<br>− counseling/case management<br>− general support<br>− training of the care receiver<br>− multi-component interventions<br>− miscellaneous interventions	− burden<br>− depression<br>− subjective wellbeing<br>− ability/knowledge	− psycho-educational interventions had significant effects on burden (-0.15 [95% CI -0,25,-0.04] t-2.76), depression (-0.27, [95% CI -0.41, -0.13], t-3.88), subjective wellbeing (0.24 [95% CI 0.04, 0.44] t2.35), and ability/knowledge (0.46, 95%CI 0.28, 0.64, t4.90)<br>− cognitive-behavioral therapy had significant effects on burden (-0.36 [95% CI -0,73,-0.01] t-1.89), depression (-0.70 [95% CI -1.10, -0.30] t-3.45), No significant effects were found on subjective wellbeing (0.37 [95% CI -0.27, 1.01] t1.13) and ability/knowledge (1.12 [95%CI -0.23, 1.62] t1.62)<br>− counseling/ case management had significant and moderately sized effects on burden (-0.50 [95% CI -0.86,-0.14], t-2.73). No significant effects were found on depression (-0.20 [95% CI -0.63, 0.23] t-0.92), subjective wellbeing (0.42 [95% CI -0.01, 0.85] t1.93) and ability/knowledge (0.43 [95%CI -0.01, 0.86] t1.94)<br>− supportive interventions had significant effects on subjective wellbeing (based on 1 study, 2.03 [95% CI 1.36, 2.70] t5.95). No significant effects were found for effects on burden (0.01 [95% CI -0.33, 0.35] t0.07), depression (0.05 [95% CI -0.68, 0.78] t0.13) and ability/knowledge (0.29 [95%CI -0.03, 0.61] t1.77)<br>− training of the care recipient had no significant effects on burden (-0.17 [95% CI -0.60, 0.27] t-0.76), depression (0.01 [95% CI -0.56, 0.58] t0.03), subjective wellbeing (0.42 [95% CI -0.18, 1.01] t1.36) and ability/knowledge (-0.12 [95%CI -0.78, 0.49] t-0.37)<br>− multi-component interventions had no significant effects on burden (-0.03 [95% CI -0.11, 0.05] t-0.74), depression (-0.10 [95% CI -0.26, 0.06] t-1.19), subjective wellbeing (-0.13 [95% CI -0.68, 0.41] t-0.48,) and ability/knowledge (0.55 [95%CI -0.55, 1.55] t0.93,)<br>− miscellaneous interventions: no significant effects on burden (-0.20 [95% CI -0.42, 0.02] t-1.76), depression (0.12 [95% CI -0.15, 0.38] t0.86), subjective wellbeing (0.37 [95% CI -0.09, 0.83] t1.58), ability/knowledge (0.35 [95%CI -0.13, 0.82] t0.82)	− longer interventions were more likely to improve depression<br>− a higher percentage of women showed more improvements in depression and knowledge/abilities, less improvement in subjective wellbeing	Caregiver interventions have significant but small effects on reducing burden, depression, subjective wellbeing, ability/knowledge and care recipient symptoms. Psychoeducational interventions have the broadest effects, but only if they call for active participation

**Thompson et al. (2007)**	Information and support interventions including:<br>− technology-based interventions<br>− group-based interventions<br>− individual-based interventions	Various including e.g.:<br>− information<br>− perceived social support<br>− support satisfaction<br>− depression<br>− burden<br>− self-efficacy	− technology-based interventions: no significant effects found on depression (WMD 0.62 [95%CI -1.98, 3.22] p=0.64)<br>− group-based interventions: psychoeducational interventions had significant effects on caregiver depression: (-0.71 [95% CI -0.95,-0.46] p<0.00001). No significant effects were found on burden (-2.15 [95% CI -5.97,1.66] p=0.27). Support intervention had no significant effects on burden (-0.40 [95% CI -5.69, 4.90] p=0.88)<br>− individual (psycho-educational) interventions: no significant effects were found on depression (-0.21 [95% CI -0.61, 0.20] p=0.32) and on self-efficacy (0.37 [95% CI -0.28, 1.02] p=0.26)	No effects for participant and intervention characteristics were reported based on an analysis	There is little evidence that interventions aimed at supporting and/or providing information to caregivers of people with dementia are uniformly effective
**Van 't Leven et al. (2013)**	Most programs consist of multiple treatment components including:<br>− information<br>− psycho-education<br>− skills training<br>− coping strategies for the caregiver<br><br>Also self-management support interventions for persons with dementia were included viz:<br>− information, training for activities of daily life, walking or exercise, and environmental adaptation	− mood<br>− burden<br>− competence<br>− quality of life<br><br>(Also outcomes of persons with dementia were studied but not included in our meta-review)	− mood; 2 studies found significant positive effects, 1 study found a positive effect only on 18 months; 1 study a positive trend; 5 studies did not show statistically significant effects<br>− perceived burden and competence; 13 studies showed significant positive effects (not at all follow up points), 4 studies did not show statistically significant effects<br>− quality of life (n=10); 7 studies found significant positive effects, 3 studies did not show statistically significant effects	No effects for participant and intervention characteristics were reported based on an analysis	Dyadic psychosocial programs are effective, but the outcomes for the person with dementia and caregiver vary
**Vernooij-Dassen et al. (2011)**	Cognitive reframing interventions including e.g.:<br>− cognitive behavioral therapy<br>− skills building/training<br>− coping/behavior management	− anxiety<br>− depression<br>− stress<br>− carer burden<br>− coping or self-efficacy<br>− quality of life	− change in anxiety (SMD-0.21 [95%CI -0.39, -0.04] p= 0.02), depression (SMD -0.66 [95%CI -1.27, -0.05] p= 0.03) depression (subgroup analysis)SMD -0.24 [95% CI 0.42, -0.07]) and stress/ distress related to caregiving (SMD -0.24 [95% CI -0.40, -0.07] test overall effect p=0.006) indicated a significant benefit from treatment<br>− no significant benefit from treatment was found on carer burden (SMD -0.14 [95%CI -0.32, 0.03], p=0.12), coping/ self-efficacy (SMD 0.64 [95% CI -0.17, 1,45] p=0.12) and RMBPC (SMD -0.21 [95%CI 0.45, 0.03] p=0.09)	No effects for participant and intervention characteristics were reported based on an analysis	Cognitive reframing for family carers of persons with dementia showed beneficial effects over usual care for psychological morbidity (anxiety, depression) and (dis)stress. No effects were found for coping or self-efficacy, carer burden and reaction to the relative’s behavior.
