# Supplementary material for: The effectiveness of interventions in supporting self-management of informal caregivers of people with dementia; a systematic meta review
Source: BMC Geriatr. 2015 Nov 11;15:147. doi: 10.1186/s12877-015-0145-6 (PMC4642777; doi:10.1186/s12877-015-0145-6)
Supplement: Additional file 7: — Evidence for self-management support interventions reported by the included reviews on outcome. (PDF 116 kb) [file 12877_2015_145_MOESM7_ESM.pdf]

Additional file 7. Evidence for self-management support interventions reported by the included reviews on outcome

			Outcomes														
	Review	Intervention as described by the authors	Burden	Depression	Wellbeing	Ability/ knowledge	Coping skills	Self-efficacy	Decision Making confidence	Anxiety	Stress or distress	RMBPC[1]	Quality of life	Social outcomes	Mood	Health	Sense of competence
**Relationship with family**	Mantovan et al. (2010)	- Case and care management	**+** (1)				**+** / **-** (1)										
	Parker et al. (2008)	- Support interventions	**+** (2)*/ **0** (5)														
	Peacock & Forbes (2003)	- Case-management interventions		**0** (1)													
		- Psychotherapy															
	Pinquart & Sorensen (2006)	- Counseling/case management*	**+** (4)	**0** (3)	**0** (3)	**0** (3)											
**Psychological wellbeing**	Chien et al. (2011)	- Caregiver support group interventions*	**0** (24 ES)	**+** (17 ES)	**+** (19 ES)									**+** (13)			
	Mantovan et al. (2010)	- Psychotherapeutic interventions	**+** (2)/ **0** (1)	**+** (3)						**0** (2)							
		- Support interventions	**0** (1)	**+** (1)/ **0** (1)						**0** (1)							
	Pinquart & Sorensen (2006)	- Cognitive behavioral therapy*	**+** (9)	**+** (11)	**0** (1)	**0** (3)											
		- Support*	**0** (4)	**0** (2)	**+** (1)	**0** (3)											
	Vernooij-Dassen et al. (2011)	- Cognitive reframing interventions*	**0** (4)	**+** (6)			**0** (4)	**0** (4)		**+** (4)	**+** (4)	**0** (3)					
**Techniques to cope with memory change**	Mantovan et al. (2010)	- Trainingsprogram	**+** (1)/ **0** (1)	**0** (1)	**0** (1)	**0** (1)	**+** (1)								**+** (1)		**+** (1)
	Pinquart & Sorensen (2006)	- Training of care recipient*	**0** (6)	**0** (4)	**0** (3)	**0** (2)											
**Information**	Boots et al. (2013)	- Internet-based supportive interventions	**+** (2)/ **0** (2)	**+** (2)/ **–** (1)	**–** (1)		**0** (1)	**+** (4)	**+** (1)	**–** (1)	**+** (1)		**0** (1)/ **–** (1)			**0** (1)	**+** (1)
	Mantovan et al. (2010)	- Psychoeducation interventions	**+** (3)	**+** (1)/ **0** (1)	**+** (1)	**+** (1)	**+** (1)	**0** (1)									**+** (1)

	Marim et al. (2013)	- Educational programs (analysis of sensitivity)*	**+** (4)														
	Parker et al. (2008)	- Psycho-educational interventions*	**0** (7)	**+** (4)	**+** (5)			**0** (4)								**0** (3)	
	Peacock & Forbes (2003)	- Education interventions		**0** (2)													
		- Computer-networking intervention							**+** (1)								
	Pinquart & Sorensen (2006)	- Psychoeducation*	**+** (42)	**+** (32)	**+** (13)	**+** (34)											
	Thompson et al. (2007)	- Information and support interventions (technology-based interventions)*		**0** (3)													
		- Information and support interventions (group-based interventions)*	**0** (3)	**+** (5)													
		- Information and support interventions (individual-based interventions)*		**0** (7)				**0** (2)									
**Multi-component**	Mantovan et al. (2010)	- Multimodale interventions	**+** (1)/ **0** (1)	**+** (2)/ **0** (1)	**0** (1)	**0** (1)											
	Parker et al. (2008)	- Multi-component interventions	**+/0** (14)	**+/0** (14)	**0** (1)	**0** (1)							**+** (1)				
	Pinquart & Sorensen (2006)	- Multi-component interventions*	**0** (10)	**0** (8)	**0** (2)	**0** (2)											
		- Miscellaneous interventions*	**0** (11)	**0** (6)	**0** (1)	**0** (1)											
	Van 't Leven et al. (2013)	- Dyadic interventions with multiple treatment component	**+**(13)/ **0**(4)										**+** (7)/ **0** (3)		**+** (3)/ **0** (6)		**+** (13)/**0**(4)

+ significant effect for the intervention on the outcome reported by the included review
– significant reversed effect for the intervention on the outcome reported by the included review
0 no (significant) effect for the intervention on the outcome reported by the included review
( ) number of underlying studies

***combined effect of the intervention on the outcome reported by the included review**
[1]Revised Memory and Behavior Problem Checklist
